# A novel RON splice variant lacking exon 2 activates the PI3K/AKT pathway via PTEN phosphorylation in colorectal carcinoma cells

**DOI:** 10.18632/oncotarget.16603

**Published:** 2017-03-27

**Authors:** Yu Ling, Yeye Kuang, Lin-Lin Chen, Wei-Feng Lao, Yao-Ru Zhu, Le-Qi Wang, Da Wang

**Affiliations:** ^1^ Department of Colorectal Surgery, Sir Run Run Shaw Hospital, Zhejiang University of Medicine, Hangzhou, Zhejiang 310016, People's Republic of China; ^2^ Biomedical Center, Sir Run Run Shaw Hospital, Zhejiang University School of Medicine, Hangzhou, Zhejiang 310016, People's Republic of China

**Keywords:** RON, splicing variants, PTEN, PI3K/AKT pathway, colorectal cancer cell

## Abstract

Abnormal expression of the Recepteur d'Origine Nantais (RON) receptor tyrosine kinase is accompanied by the generation of multiple splice or truncated variants, which mediate many critical cellular functions that contribute to tumor progression and metastasis. Here, we report a new RON splice variant in the human colorectal cancer (CRC) cell line HT29. This variant is a 165 kda protein generated by alternative pre-mRNA splicing that eliminates exon 2, causing an in-frame deletion of 63 amino acids in the extracellular domain of the RON β chain. The deleted transcript was a single chain expressed in the intracellular compartment. Although it lacked tyrosine phosphorylation activity, the RONΔ165^E2^ variant could phosphorylate phosphatase and tensin homolog (PTEN), thereby activating the PI3K/AKT pathway. In addition, *in vitro* and *in vivo* experiments showed that the RONΔ165^E2^ promoted cell migration and tumor growth. Finally, in an investigation of 67 clinical CRC samples, the variant was highly expressed in about 58% of the samples, and was positively correlated with the invasive depth of the tumor (P < 0.05). These results demonstrate that the novel RONΔ165^E2^ variant promoted tumor progression while activating the PI3K/AKT pathway via PTEN phosphorylation.

## INTRODUCTION

Recepteur d'Origine Nantais (RON), a tyrosine kinase receptor, is a member of the Met proto-oncogene family [1]. Members of this family share a similar structure consisting of an extracellular ligand-binding domain, a hydrophobic membrane-spanning domain, and a large cytoplasmic portion with catalytic function [2, 3]. Met is the receptor for hepatocyte growth factor (HGF) [4], and the interaction of both proteins activates multiple intracellular signaling pathways involved in muscle and liver formation, cell proliferation, morphogenesis, and motility [5]. Aberrant expression, constitutive activation, and point mutation in Met are implicated in the development and progression of a number of tumors [5, 6]. The gene encoding RON maps to region 3p21 on the human chromosome, encompassing 20 exons [7]. Mature RON is a 180 kda heterodimeric protein composed of a 40 kda extracellular α chain and a 150 kda transmembrane β chain linked by disulfide bonds, which originated from proteolytic cleavage of a common precursor [8]. The specific ligand for RON is the macrophage-stimulating protein (MSP) [2], also known as HGF-like protein. Binding of MSP to the extracellular domain of RON stimulates its intrinsic tyrosine kinase activity and phosphorylation of the docking site, which is recognized by multiple SH2 domain-containing transducer and adapter proteins. Multiple intracellular signaling cascades are involved in the activation of RON-transduced signals, including the ras/mitogen-activated protein kinase (MAPK), phosphatidyl inositol-3 kinase (PI3K)/AKT, and focal adhesion kinase pathways, which elicit distinctive biological responses such as cell dissociation, motility, and polarized growth [9].

RON is expressed in several epithelial tissues, in certain types of tissue macrophages, and in some erythroleukemia cell lines [2]. Studies from the past 20 years have shown that it is required for embryonic development and is involved in the innate immune response against pathogen-induced inflammatory reactions [10]. However, recent *in vitro* and *in vivo* studies have shown that RON expression or activation is altered in epithelial carcinomas including lung, colon, and breast cancers [6, 11–15], indicating that abnormal activation of this receptor may play a role in the progression of certain epithelial cancers. Different isoforms generated by alternative splicing is one of the mechanisms underlying RON activation in cells, which significantly increases its oncogenic activities [13, 16–21]. Recently, we identified a novel RON isoform derived from human colorectal cancer (CRC) tissues, which lacks exon 2, encodes 63 amino acids in the extracellular domain of the RON β chain, and has a molecular weight of 165 kda. We named this variant RONΔ165^E2^ and observed that it exists as a single-chain protein located in the cytoplasm. The RONΔ165^E2^ variant lacked tyrosine phosphorylation activity and constitutively hyperactivated the PI3K/AKT pathway via phosphatase and tensin homolog(PTEN) phosphorylation, which induced the invasive phenotype in epithelial cells.

## RESULTS

### Detection of a new splice variant in human CRC cell lines

**Figure 1:**
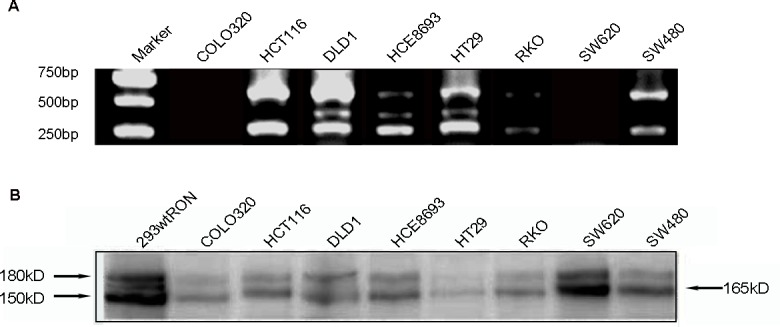
Detected the novel RON variant in CRC cell lines **(A)**, in RT-PCR assay using the pair 3 primers, we observed an additional short band (about 350 bp) which missing 200 bp compared to the wtRON in HT29, HCE8693, DLD1 cell lines. **(B)**, Western blotting result: utilizing the antibody against the RON C-terminal of β chain, we identified an about 165 kD band in HT29.

In our current study, we chose eight human CRC cell lines: HCE8693, SW480, RKO, COLO320, SW620, HCT116, HT29, DLD1 to detect the RON variant. To this end, we extracted total RNA from the CRC cell lines and used a Superscript Preamplification Kit for reverse transcription (RT) (see Material and Methods). PCR was conducted on the products of the RT reactions using the primer pair 3. The results are shown in Figure 1A. While the product of wild-type RON (wtRON) was about 550 base pairs (bp), we also found an additional short segment in HCE8693, HT29, and DLD1 cells, in addition to a 250 bp segment that lack exons 2 and 3 [22]. The product was close to 350 bp, and may lack approximately 200 bp nucleotides (nt) between exons 2 and 5. To further study expression of the RON variant in cancer cells, we performed Western blot analysis with an anti-RON(H160) antibody to detect RONΔ165^E2^ expression in the eight CRC cell lines; wtRON was used as the positive control. The results are shown in Figure 1B. The wtRON showed two bands at 180 kDa and 150 kDa. In the HT29 cell line, there was a band near 165 kDa that may represent the novel RON variant. However, due to the lack of a specific antibody against RONΔ165^E2^ as well as the similar molecular weight of several RON isoforms, it was difficult to distinguish the new variant from other variants. Than, we isolated the total RNA from HT29 cells using TRIzol (Life Technologies). RT-PCR was conducted by using the primer pair 1. The 700bp, 500bp and 400bp PCR fragments covering the nucleotides sequence from 1139nt to 1799nt were amplified. The 500bp fragment was then subcloned into the pGEMT-Easy vector (Promega) for subsequent studies.

### Expression of RONΔ165^E2^ cDNA in human embryonic kidney 293 cells

**Figure 2:**
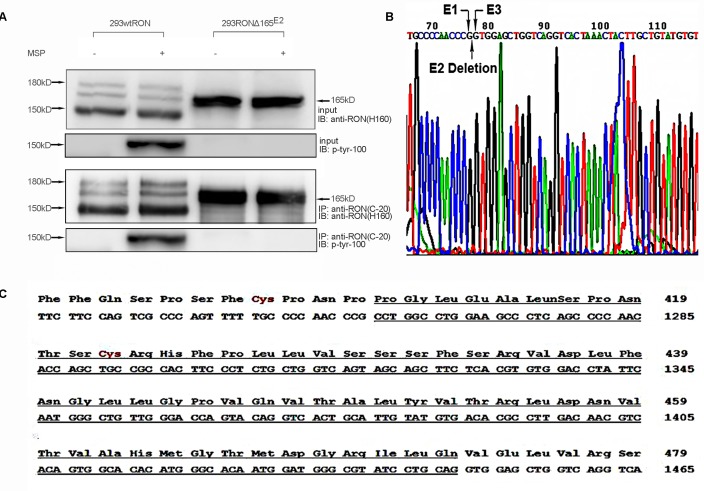
Characteristics of the novel RON variant **(A)** In stable wtRON or RONΔ165^E2^-transfected HEK293 cells lysates, by utilizing the antibody RON(C-20) for CO-IP efficiently pulled down the corresponding target protein RON, we used the RON antibody (H160) to detect the expression of the RONΔ165^E2^. A single-chain protein was clearly the novel RON variant. The antibody to phosphorylated tyrosine residues p-Tyr-100 was used to explore the activation level of RONΔ165^E2^. As shown, there was increased phosphorylation in HEK293 wtRON cells after treatment with MSP (2 nM), while there was very little phosphorylation in cells expressing RONΔ165^E2^. 20% of total cell lysates were subjected to western blotting with the indicated antibodies as inputs. **(B)** PCR amplification product from primer pair 1 was sequenced. Compared to the wtRON cDNA sequence, the novel variant lacked 189 bp, which indicated the deletion of exon 2. **(C)** Partial protein translation according to the codon of wtRON cDNA. The deleted 63 amino acids and 189 nt in RONΔ165^E2^ were underlined with a solid line.

To explore the bio-characteristics of the new RON variant, we isolated and subcloned the short fragment into the pGEMT vector, which was sequenced by GenScript, Inc. (NanJing, China); the sequence is shown in Figure 2B. The sequencing results showed that the new variant lacks 189 nt between positions 1259 and 1447 compared with the wtRON sequence. The missing region corresponded to exon 2, which resulted in the deletion of 63 amino acids in the extracellular domain of the β chain (Figure 2C). Because the speculated molecular weight of this new variant was similar to RON165, a RON variant missing exon 11, we named it RONΔ165^E2^ to reflect the deletion of exon 2. Then we cloned full-length RONΔ165^E2^ cDNA and constructed the pcDNA4/HisMaxC RONΔ165^E2^ plasmid (see Material and Methods), which was transfected into human embryonic kidney 293 (HEK293) cells using the FuGENE-HD transfection reagent (Roche, Basel, Switzerland). Cells were selected for 2 weeks using 100 g/ml zeocin. The expression of RONΔ165^E2^ was determined by Western blotting with antibody RON(H160) against the RON receptor. Cells stably expressing RONΔ165^E2^ were pooled and used in subsequent experiments, and HEK293 cells expressing wtRON were used as the control. Cell lysates were precipitated by RON C terminus antibody. Proteins in the immunocomplexes were then extracted in SDS sample buffer and used for immunoblotting to identify interacting proteins. After western blotting with the antibody RON(H160), two bands were detected at 180 kDa and 150 kDa from wtRON-transfected cells in Figure 2A. However, HEK293 cells expressing RONΔ165^E2^ only showed one band at 165 kDa, indicating that the RONΔ165^E2^ protein was a single-chain protein with an uncleaved β chain. Meanwhile, Western blotting with an antibody against p-Tyr-100 showed that the RON variant had no phosphorylation activity in the presence or absence of MSP stimulation in Figure 2A. In HEK293 cells expressing wtRON, the tyrosine phosphorylation of wtRON significantly increased after the addition of MSP compared to basal levels.

### RONΔ165^E2^ protein was expressed in the intracellular compartment

**Figure 3:**
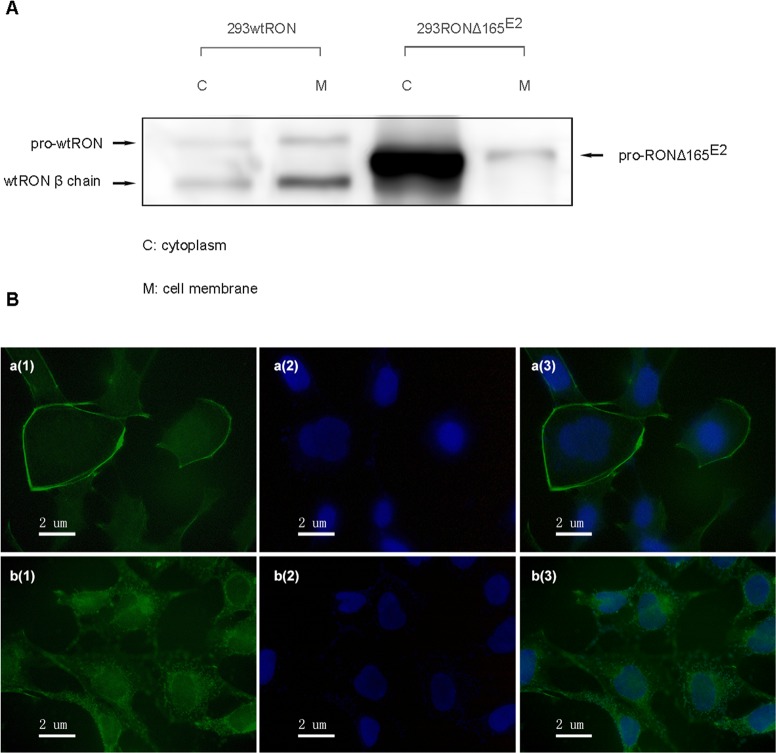
Cellular localization of RONΔ165^E2^ **(A)** Using a Cell Surface Protein Isolation Kit, the cytoplasmic and cell-surface proteins were separated by SDS-PAGE and incubated with an antibody against RON (H160). In HEK293 wtRON cells, the pro-RON (180kD) and mature RON protein (150 kDa) both existed on the cell surface, while in HEK293 RONΔ165^E2^ cells, the variant protein was expressed in the cytoplasm. **(B)** Detection of RON expression by immunofluorescence staining in both HEK293 wtRON and HEK293 RONΔ165^E2^ cells. a(1), b(1) were respectively on behalf of the location about wtRON protein, RONΔ165^E2^ protein which were dyed green by FITC. a(2), b(2) nuclei were counterstained with DAPI (blue). a(3) was the merged picture of a(1) and a(2), while b(3) was the merged picture of b(1) and b(2).

Based on our current knowledge about RON variants in the carcinogenesis of epithelial cancers, RONΔ165^E11^, RONΔ155, RONΔ160^E2E3^ showed a single-chain form and were present in the cytoplasm without the mature β chain [16]. According to the aforementioned Western blotting results, we hypothesized that the RONΔ165^E2^ protein may be localized in the intracellular compartment. To confirm our hypothesis, a cell surface labeling experiment was conducted in HEK293 cells expressing wtRON and RONΔ165^E2^ to determine if RONΔ165^E2^ protein is expressed on the cell surface or in the cytoplasm. Cells were labeled with EZ-Link Sulfo-NHS-SS-Biotin and subsequently lysed in a mild detergent, after which the labeled proteins were isolated with NeutrAvidin Agarose. The flow-through fraction contained the cytoplasmic proteins and the eluent from the agarose contained the cell surface proteins. The two kinds of cell lysates were subjected to SDS-PAGE and Western blotting using the RON (H160) antibody. The results showed that the wtRON protein was expressed in two forms, whereas RONΔ165^E2^ was only expressed in the cytoplasm as a single band (Figure 3A). Immunofluorescent staining using antibodies against the RON α chain confirmed that the RONΔ165^E2^ protein was localized in the cytoplasm (Figure 3B).

### The PI3K/AKT/mTOR pathway was constitutively activated in HEK293 RONΔ165^E2^ cells

**Figure 4:**
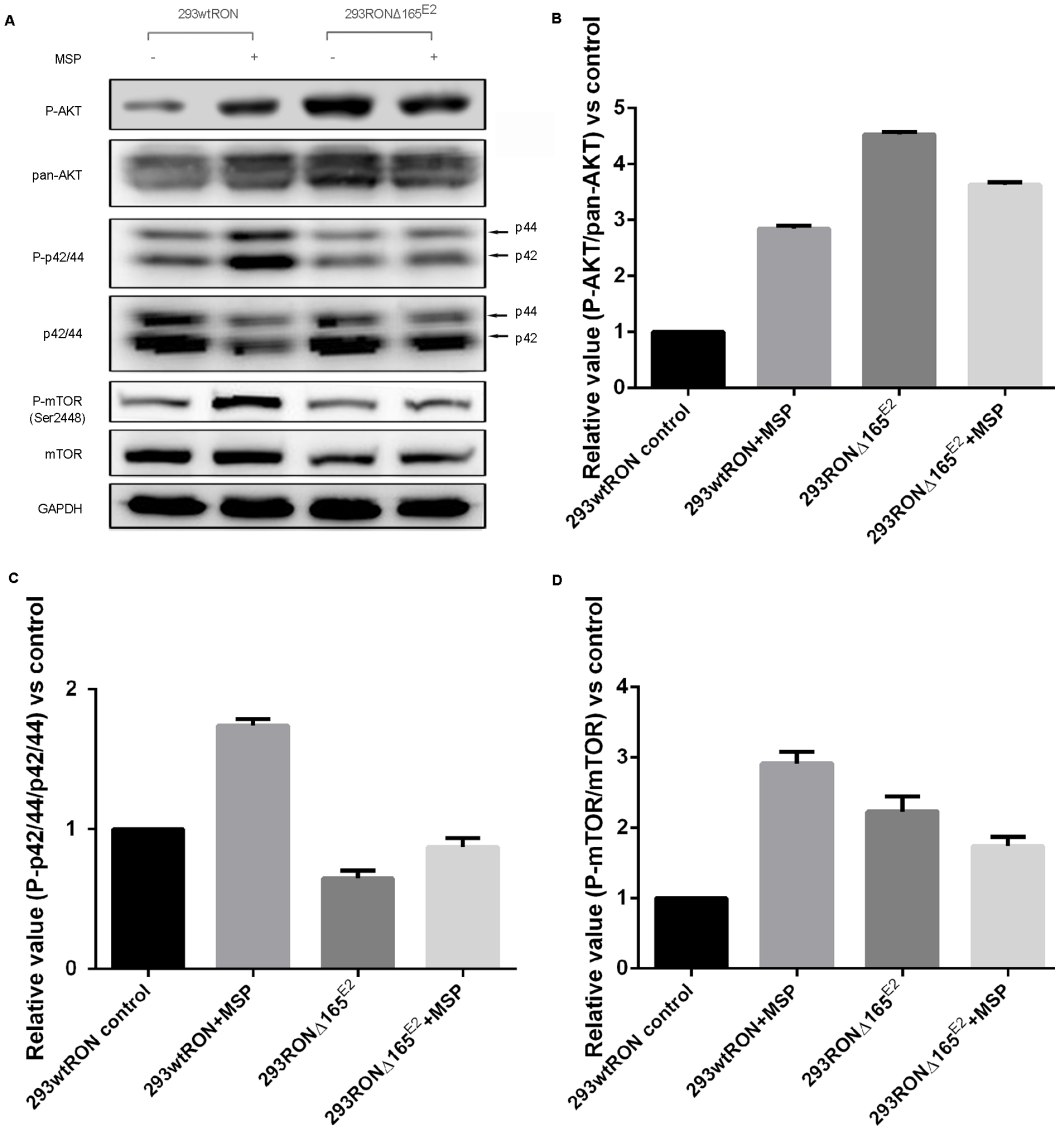
Constitutive activation of the PI3K/Akt pathway in 293 RONΔ165^E2^ cells **(A)** Western blotting results on the activation of signaling proteins involved in the PI3K/Akt and MAPK pathways. **(B)** The relative ratio of phosphorylated to total Akt expression in HEK293 wtRON cells was significant after MSP stimulation. While there was no meaningful change in the relative ratio of phosphorylated to total Akt expression in HEK293 RONΔ165^E2^ cells with or without MSP, it was significantly higher than that in HEK293 wtRON cells even with MSP stimulation (p < 0.05). **(C)** Relative value of p-p44/42/p44/42 in HEK293 wtRON cells was significantly increased after MSP stimulation. While there was no meaningful change in HEK293 RONΔ165^E2^ cells with or without MSP, it was significantly lower than that in HEK293 wtRON cells (p < 0.05). **(D)** Relative ratio of p-mTOR/mTOR was increased in HEK293 RONΔ165^E2^ cells compared with HEK293 wtRON cells (p < 0.05). Compared with HEK293 wtRON cells in the quiescent state, the level of the relative p-mTOR/mTOR ratio was significantly increased after they were treated by MSP. MSP had no effect on the level of mTOR activation in HEK293 RONΔ165^E2^ cells.

Aberrant activation of signaling pathways involved in proliferation or development is commonly associated with cancer progression. PI3K/AKT and MAPK pathways are two independent signaling pathways that mediate the antiapoptotic actions of MSP on epithelial cells, which are also usually two downstream pathways of the RON receptor tyrosine kinase. We conducted Western blotting to investigate changes in these signal transduction pathways in HEK293 cells expressing RONΔ165^E2^; HEK293 cells expressing wtRON were used as the positive control. The results are summarized in Figure 4. In HEK293 cells expressing wtRON, most of the signaling proteins such as AKT and Erk1/2 were activated, meanwhile increased markedly after MSP stimulation. Compared to HEK293 wtRON cells not treated with MSP, in untreated HEK293 RONΔ165^E2^ cells, the relative activation rate of AKT and mTOR significantly increased (p < 0.05), whereas the MAPK pathway was slightly inhibited. In accordance with our previous results, there were no significant differences between the untreated or MSP-treated group in HEK293 RONΔ165^E2^ cells, indicating that the RON variant was not interaction with MSP.

### RONΔ165^E2^ increased PTEN phosphorylation

**Figure 5:**
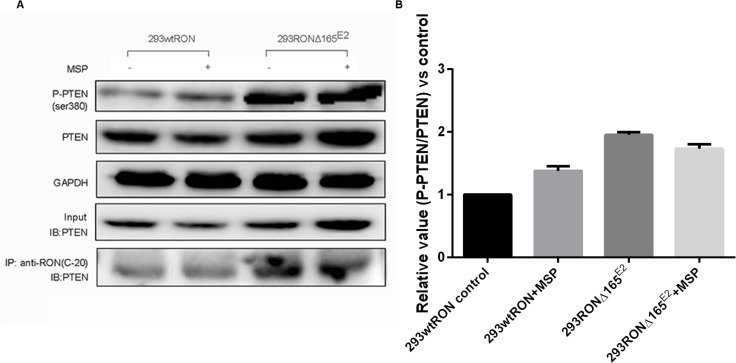
PTEN lipid phophatase activity was decreased in HEK293 RONΔ165^E2^ cells despite the upregulated PTEN protein expression **(A)** western blotting results using the antibodies against PTEN, P-PTEN(Ser380) indicated higher level in HEK293 RONΔ165^E2^ cells compared to the HEK293 wtRON cells, p < 0.05. After co-immunoprecipitation with the antibody RON(C-20), western blotting with the antibody specific to PTEN showed that the RONΔ165^E2^ protein had an obviously interaction with the PTEN protein in cytoplasm. 20% of total cell lysates were subjected to westernblotting with the PTEN antibody as input. **(B)** compared with the control group, in HEK293 RONΔ165^E2^ cells it showed an increasing phosphorylation level of PTEN without the impact of MSP stimulation.

We speculated that PTEN, an essential tumor suppressor gene which encodes a phosphatase protein that antagonizes the PI3K/AKT/mTOR antiapoptotic pathway, may regulate the pathway downstream of RONΔ165^E2^. Western blotting was used to detect phosphorylated and total PTEN expression. In HEK293Δ165^E2^ cells, the relative phosphorylation ratio of PTEN was higher than in HEK293 wtRON cells (p < 0.05). As confirmation of these results, we used Western blotting with the antibody specific to PTEN after co-immunoprecipitation with RON(C-20) to show the interaction among PTEN and the novel RON variant. As shown in Figure 5, the amount of PTEN binding to the variant RON protein was highly increased in HEK293 RONΔ165^E2^ cells.

### RONΔ165^E2^ increased the motility capacity of HEK293 cells

**Figure 6:**
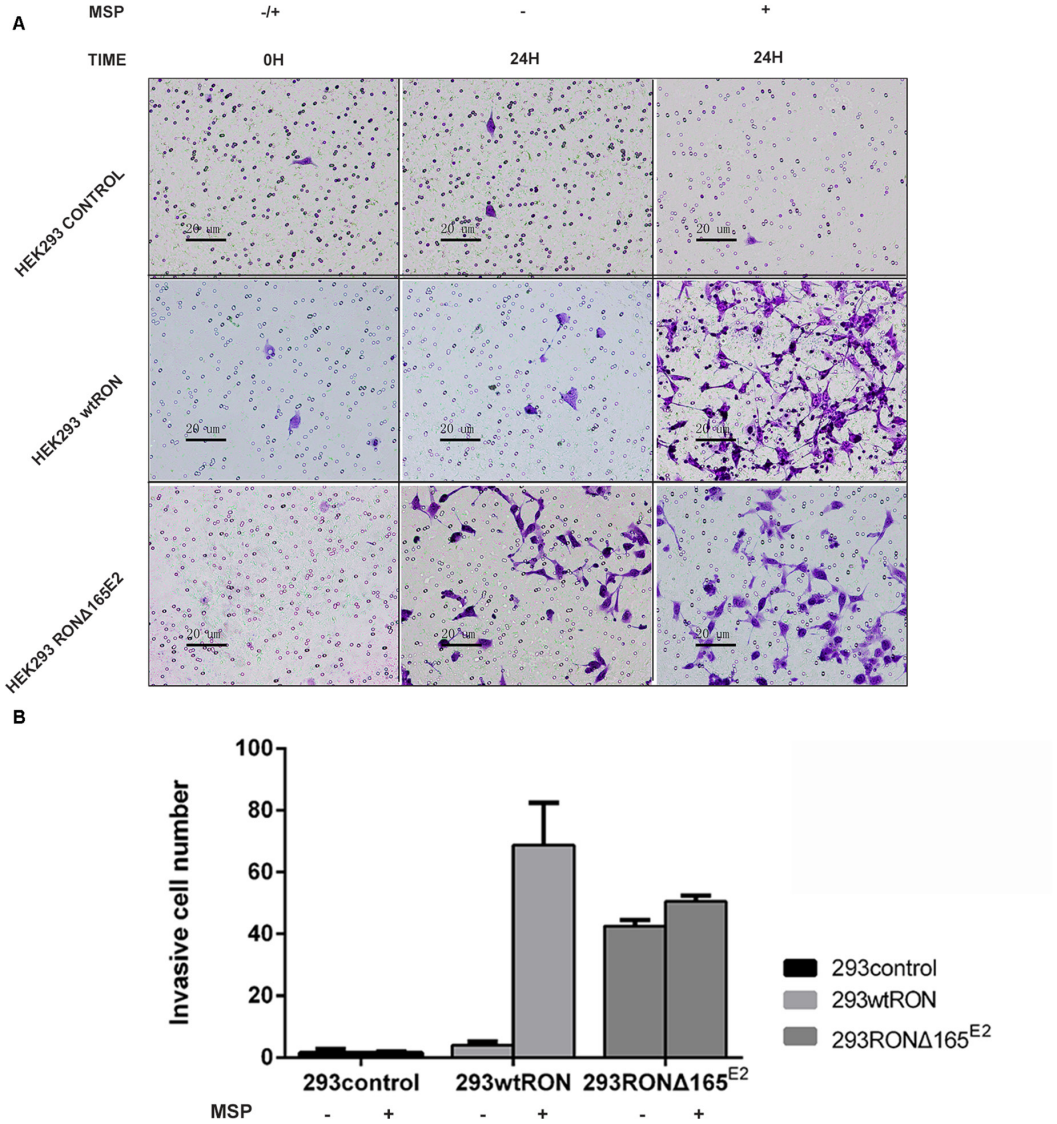
RONΔ165^E2^ improved the motility ability of HEK293 cells (×200) **(A)** the number of migration cells after observation 24 hours with or without MSP (2 nM). **(B)** compared with HEK293 empty vector control group, HEK293 wtRON group, the mean migration cells were increased in 293 RONΔ165^E2^ cells, p < 0.05. All experiments were repeated three times.

**Figure 7:**
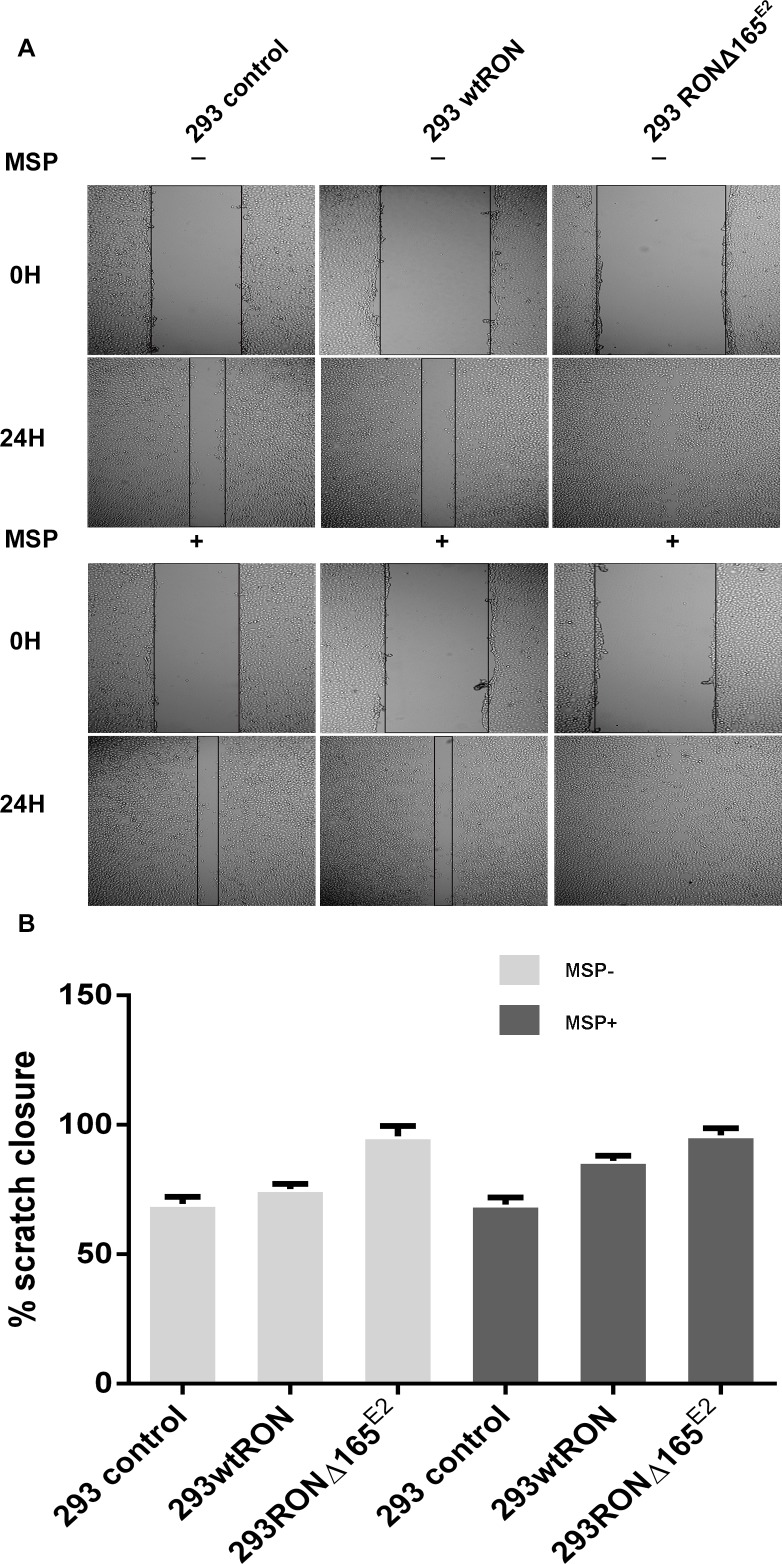
RONΔ165^E2^ improved the motility of the HEK293 cells (×100) **(A)** in the wound healing assay, all three groups were treated with or without MSP, respectively. After 24 hours, all groups were photographed. **(B)** the motion ability of HEK293 wtRON cell increased after MSP stimulation compared with its negative group, p < 0.05. Compared with the HEK293 control group and HEK293 wtRON group, the ratio of scratch closure improved in HEK293 RONΔ165^E2^ group, p < 0.05.

In the transwell assay, the number of cells that migrate to the bottom chamber of the transwell chamber can reflect the motility capacity of the cells. Our experiment showed that the invasive ability of HEK293 RONΔ165^E2^ cells was significantly higher than that of the empty vector control and HEK293 wtRON cells. Similarly, the number of cells that penetrated the matrigel in the transwell chamber cells increased after MSP stimulation in HEK293 wtRON cells, whereas there was no change in HEK293 RONΔ165^E2^ cells (Figure 6A, 6B). The results of the wound-healing assay showed that after scratching for 24 h, the migration ability of the HEK293 RONΔ165^E2^ cells was considerably higher than that of HEK293 wtRON cells and the empty vector control (p < 0.05). Besides, the cell migration capacity of the wtRON group was meaningfully enhanced after MSP stimulation. However, we found the ability of the new variant transfected cells to move unaffected after MSP stimulation (Figure 7).

### Overexpression of RONΔ165^E2^ promoted tumor formation in mice

**Figure 8:**
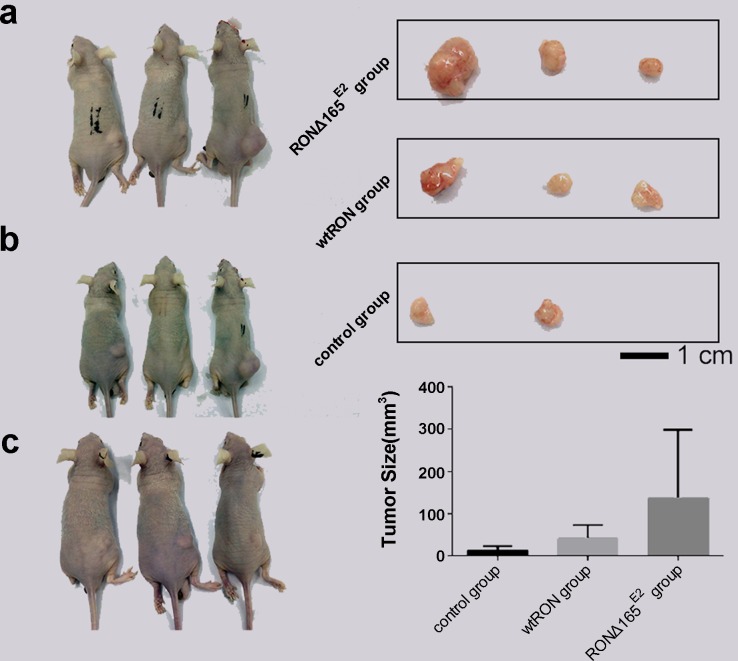
Biological characteristic of RONΔ165^E2^ *in vivo* **(a)** represented the groups who were treated with the SW48 RONΔ165^E2^ cells by subcutaneously in the dorsal flank. **(b)** represented the groups who were treated with the SW48 wtRON cells by subcutaneously in the dorsal flank. **(c)** represented the groups who were treated with the empty vector control by subcutaneously in the dorsal flank. The top rights in Figure 8 were the solid tumors from the corresponding treatment groups. The lower rights in Figure 8 were the mean tumor sizes of each group mice after being implanted tumor cells for 30 days. Compared with the control group and the SW48 wtRON group, the variant RON confirmed itself the significant characteristic to promote tumor growth.

We constructed a tumor cell line by transfecting the plasmid expressing the novel RON variant cDNA or prototype RON cDNA in SW48 cells; empty vector served as the negative control. Then to explore the bio-characteristic of RONΔ165^E2^ *in vivo*, we used nude mice subcutaneously infected with the transfected SW48 as models. The growth of subcutaneous-transplanted tumors was observed daily. After tumor formation, tumor volume was measured every 3 days. After the tumor cells had been implanted for 30 days or the tumor had reached a volume of 1000 mm^3^, all of the mice were sacrificed and the tumors were removed. The results showed that in the SW48 RONΔ165^E2^ cell group, the mean tumor size was significantly enhanced compared with the SW48 wtRON cell group and empty vector control (p < 0.05) (Figure 8).

### A clear association between RONΔ165^E2^ amplification and invasive depth of the tumor

**Figure 9:**
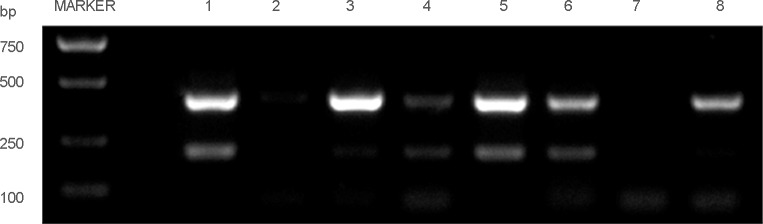
Part of the RT-PCR results among the clinical CRC specimens Note: the 350 bp band was the wtRON DNA fragment, the 200 bp band was the RON variant DNA fragment.

Finally, we investigated the relationship between the RONΔ165^E2^ variant and the clinicopathologic characteristics of the CRC tumor tissues. To this end, we collected 67 CRC samples from the Sir Run Run Shaw Hospital (Zhejiang, China). Total RNA was extracted from the specimens and PCR was conducted on the products of the RT reactions using the oligomer pair 2 in Figure 9, and details of the specimens' characteristics and statistical results are shown in Table 1. RONΔ165^E2^ was highly expressed in about 58% of the specimens. In addition, its expression correlated with the invasive depth of the tumor (p < 0.05).

**Table 1:** Relationship between the variant RON deletion exon 2 and clinicopathologic features of CRC patients

Characteristic	SummaryCase no.(%)	Ron200^b^Expression		Ron200-350^c^Expression		Ron200+350^d^Expression	
		Caseno.(%)	P value^a^	Caseno.(%)	P value^a^	Caseno. (%)	P value^a^
**Age**			0.918		0.978		0.932
≤58 yrs	22(32.8)	13(19)		2(3)		11(16)	
>58 yrs	45(67.2)	26(39)		4(6)		22(33)	
**Gender**			0.514		0.660		0.695
male	39(58.2)	24(36)		4(6)		20(30)	
female	28(41.8)	15(22)		2(3)		13(19)	
**Histologicdifferentiation**			0.756		0.501		0.935
well	48(71.6)	27(40)		4(6)		23(34)	
moderate	15(22.4)	9(13)		1(1)		8(12)	
poor	4(6.0)	3(4)		1(1)		2(3)	
**Tumor location**			0.819		0.833		0.729
colon	25(37.3)	15(22)		2(3)		13(19)	
rectum	42(62.7)	24(36)		4(6)		20(30)	
**T classification**			**0.045**		0.978		**0.046**
T1-3	22(32.8)	9(13)		2(3)		7(10)	
T4	45(67.2)	30(45)		4(6)		26(39)	
**N classification**			0.220		0.787		0.172
N0	30(44.8)	15(22)		3(4)		12(18)	
N1-3	37(55.2)	24(36)		3(4)		21(31)	
**M classification**			0.391		0.369		0.174
M0	62(92.5)	37(55)		5(7)		32(48)	
M1	5(7.5)	2(3)		1(1)		1(1)	
**Clinical stage**			0.220		0.787		0.172
I-II	30(44.8)	15(22)		3(4)		12(18)	
III-IV	37(55.2)	24(36)		21(31)		21(31)	

^a^ Chi-Square test. Bold value were high light the significant value (p<0.05).

^b^ Ron200, Electrophoresis in 200 bp ban.

^c^ Ron200-350, Electrophoresis in 200 bp ban expect the 350 bp ban.

^d^ Ron200+350, Electrophoresis in 200 bp and 350 bp ban.

## DISCUSSION

Previous studies have demonstrated seven different RON splice variants, most of which contribute to the malignant phenotype [14, 16–23]. Here, we identified a novel RON variant from the human CRC cell line HT29. To the best of our knowledge, this variant lacks exon 2, which encompasses 63 amino acids in the extracellular domain. It has a molecular weight of 165 kDa, similar to RONΔ165, which originated from a spliced mRNA transcript with an inframe deletion in exon 11 [24]; thus, we named it RONΔ165^E2^ accordingly.

To determine the biochemical properties unique to the RON splice variant, the cDNA fragment containing the deleted exon 2 was amplified from mRNA isolated from HT29 cells. Sequence analysis revealed that the fragment was derived from a RON transcript with a deletion of 189 nucleotides from 1259 to 1447 encompassing 63 amino acids. The missing fragment belonged to the Sema domain in the extracellular sequence of the RON β chain, resulting in elimination of the domain for ligand binding and receptor homodimerization upon ligand stimulation. In addition, among the missing 63 amino acids was one cysteine residue that is normally involved in intracellular disulfide bonds. This imbalance in cysteine pairs renders the uneven cysteines available for intercellular disulfide bonding with other Δ-RON partners, thereby creating oligomers. A similar mechanism has been proposed for RONΔ160^E2E3^ [22]. While wtRON protein had a heterodimeric structure under reducing and unreducing conditions [2], single-chain RONΔ165^E2^ significantly affected formation of the three-dimensional structure and caused the protein to be retained in the cytoplasm. We utilized cell-surface biotinylation assays and immunofluorescence experiments to confirm the misfolding of the Δ-RON protein in the intracellular vesicular compartment. The results were similar to those found with the RONΔ165 variant [24].

HEK293 cells express little endogenous RON protein compared to common types of colon cancer cell lines [45]. To explore the invasive ability of Δ-RON in epithelial cells, we transfected a plasmid expressing RONΔ165^E2^ or wtRON in HEK293 cells. As observed in Western blotting experiments, the variant was expressed as a single band of about 165 kda without tyrosine phosphorylation activity. A previous study showed that wtRON and its splice variants, such as oncogenic RONΔ165 and RONΔ155 [24], stimulated intracellular signal pathways by activating the tyrosine kinase domain through phosphorylation of tyrosine residues 1238 and 1239, which induced the invasive phenotype in colorectal cells. The deficiency in tyrosine kinase activity prompted us to elucidate the potential biological activities of RONΔ165^E2^. Cytoplasmic kinases are intracellular messengers that play an important role in signal transduction. Thus, we examined the effects of HEK293 cells expressing wtRON or RONΔ165^E2^ on two common downstream pathways, PI3K/AKT and MAPK, in the presence or absence of MSP [10, 26–33]. To our surprise, in HEK293 RONΔ165^E2^ cells, the PI3K/AKT/mTOR pathway was significantly activated, whereas the MAPK pathway was significantly inhibited. The PI3K/AKT/mTOR pathway promotes cell migration [34]; thus, we examined the motility ability of the HEK293 cells transfected with RONΔ165^E2^ or wtRON. In accordance with activation of the PI3K/AKT pathway, we found significant invasion and migration of HEK293 RONΔ165^E2^ cells compared to HEK293 wtRON cells. The expression of the novel RON variant was directly correlated with tumor growth *in vivo* animal models. In addition, RONΔ165^E2^ was expressed in 58% of the clinical samples investigated. These data suggest that activation of the PI3K/AKT pathway may underly the biological function of RONΔ165^E2^.

To date, the main putative negative regulator of the PI3K/AKT pathway is the lipid phosphatase PTEN [27, 35, 36], which is frequently altered in human cancer ultimately resulting in decreased or absent PTEN protein expression and activity. Human T cell acute lymphoblastic leukemia cells had constitutive hyperactivation of the PI3K/AKT pathway via PTEN phosphorylation [37]. The PTEN tumor suppressor regulates cell migration, growth, and survival by dephophorylating phosphatid inositol second messengers and signaling phosphoproteins [38]. The C-terminal catalytic regulatory domain of PTEN contains multiple putative phosphorylation sites, which could play an important role in the control of its biological activity [39]. Phosphorylation of its C-terminal domain appears to stabilize the protein by preventing its ubiquitination and proteasome degradation while decreasing its phosphatase activity [39]. In our study, the expression level of PTEN slightly increased in HEK293 RONΔ165^E2^ cells compared to HEK293 wtRON cells. However, in HEK293 RONΔ165^E2^ cells, the significant increase in phosphorylated Ser/Thr residues at the PTEN C-terminal domain (C-tail) may indicate its interaction with an intracellular variant. To evaluate the potential cooperative effects of the RON variant and PTEN, co-immunoprecipitation was performed from cell lysates with a unique RON antibody (C-20) bound to protein A/G agarose beads, and PTEN protein was detected with PTEN antibodies. We observed a significant interaction between the variant and PTEN protein in our experiment. To date, phosphorylation of the cluster in the PTEN C-tail is mainly driven by casein kinase 2 [40], Src, and s6k, whereas phosphorylation of Ser362 and Thr366 is conferred by glycogen synthase kinase 3β [29, 36, 41, 42]. As previously reported, the Src family of non-receptor tyrosine kinases is a critical component of signaling cascades initiated by tyrosine kinase-linked receptors such as the epidermal growth factor receptor, G protein-coupled receptors including the lysophosphatidic acid receptors, and steroid receptors including the estrogen receptor, due to its SH2 domain [43]. Src is overexpressed or highly activated in a number of human neoplasmas including CRC [44]. In an *in vitro* study, it was demonstrated that the Src protein tyrosine kinase participates in the regulation of PI3K/AKT pathway by altering PTEN function, which is associated with an increase in the tyrosine phosphorylation of PTEN [43]. We speculate that intracellular expression of the RON variant may impact phosphorylation of Src and PTEN by a protein-protein interaction motif such as SH2-binding sites, followed by activation of the PI3K/AKT pathway in the absence of tyrosine phospharylation. This pathogenesis needs further molecular and animal model studies.

In our carcinoma specimens collected from 67 cases, we found a high incidence of the novel variant. In addition, we found that this variant was correlated with locally advanced tumors, especially invasion of the outer serosal layer of the colon. Thus, this RON variant may affect tumor staging and the prognosis of colon cancer.

Small-molecule tyrosine kinase inhibitors or specific antibodies that target RON have been extensively studied in cancer chemotherapy because it is frequently altered in epithelial cancers, such as colon and breast cancers, and receptor splice variants are increasingly implicated in mechanisms underlying oncogenesis, metastasis, and the creation of drug-resistant phenotypes. This study identified a novel RON variant that interacts with PTEN by a mechanism that remains to be determined. The interaction led to increased PTEN phosphorylation, resulting in activation of the PI3K/AKT/mTOR pathway in HEK293 cells. We suspect that this may be one mechanism of targeted therapy failure in CRC. Our future studies will focus on the relationship between PTEN and other variants such as RONΔ160^E2E3^. We hope to find a new effective strategy for treating CRC.

## MATERIALS AND METHODS

### Ethics statement

This study was approved by the Ethics Committee of Sir Run Run Shaw Hospital, Zhejiang University of Medicine, hangzhou, People's Republic of China. All animal experiments complied with the Animal Care Guideline of Zhejiang University, hangzhou, People's Republic of China.

### Tissue collection

A total of 74 CRC tissue specimens were collected from 74 patients operated in the Sir Run Run Shaw Hospital. All patients consented to the use of their tissue for research. All specimens were confirmed as CRC by pathological diagnosis. 7 patients were excluded due to insufficient primary tumor tissue. The study included 28 females and 39 males, ranges in age from 34 to 88 years with a mean age of 64 years. The clinicopathological information is summarized in table. Tissues were frozen in liquid nitrogen, and then stored at −80°C before use.

### Chemicals and antibodies

Recombinant human MSP was from R&D Systems (Minneapolis, MN, USA). The rabbit IgG antibodies specific to phospho-PTEN (Ser380/Thr382/383), phpspho-p44/42 MAPK (Erk1/2)(Thr202/Tyr204), phospho-(Ser/Thr) AKT, pan-AKT (C67E7), the rabbit mAb to p44/42 MAPK (Erk1/2)(137F5), the rabbit mAb to phospho-mTOR (Ser2448) (D9C2) and the mouse anti-phospho tyrosine mAb (p-tyr-100) were from the company Cell Signaling, Inc. (Beverly, MA, USA). The rabbit IgG antibodies specific to the extracellular domain of human RON (H160), the C-terminal of RON(C-20), and the mouse mAb specific to PTEN were from Santa Cruz Biotechnology(Santa Cruz, CA, USA). The mouse mAb against the RON α-chain was from BD Biosciences (San Jose, CA, USA). Tyrosine kinase assay kit (non-radioactive) was from Upstate (Lake Placid, NY). The normal rabbit or mouse IgG were from Beyotime biotechnology(Beyotime, Shanghai, China).

### Reverse transcription(RT)-PCR assay and DNA sequencing

Total RNAs were extracted three times from colorectal cancer cell lines and specimens using TRIzol (Life Technologies). Reverse transcription was carried out using 2 ug of total RNAs with a Superscript Preamplification Kit (Life Technologies). PCR was conducted on the products of RT reactions. The oligomers for PCR amplification used to clone Δ-RON cDNA was designed on the basis of the RON cDNA sequences as follows:

pair 1, sense oligomer corresponding to nucleotides 1139 to 1158 (5′- ATTGACCTGCTGGACACACT-3′) (ron1-1), and antisense oligomer corresponding to nucleotides 1780 to 1799 (5′- CACACAGGGTCAGCCTTGTA-3′) (rone5r);

pair 2, sense oligomer corresponding to nucleotides 1227 to 1245 (5′- tcttccagtcgcccagttt-3′) (rone2f), and antisense oligomer corresponding to nucleotides 1553 to 1572 (5′-TCCCCAGAGGCAAAGAGTAG-3′) (RONE2R);

pair 3, sense oligomer corresponding to nucleotides 1227 to 1245 (5′- tcttccagtcgcccagttt-3′) (rone2f), and antisense oligomer corresponding to nucleotides 1780 to 1799 (5′-CACACAGGGTCAGCCTTGTA-3′) (rone5r);

The oligomers for PCR amplification used to clone GAPDH cDNA was as follows: pair 4, sense oligomer(5′-GGCTCTCCAGAACATCATCCCTGC-3′), antisense oligomer(5′-GGGTGTCGCTGTTGAAGTCAGAGG-3′).

Double-stranded DNA sequencing was performed by GenScript, Inc.(NanJing, China).

### Cell culture

The human colorectal carcinoma cell lines COLO320, SW620, RKO, HCE8693, SW480, HCT116, HT29, DLD1, SW48, the human embryonic kidney cell line HEK293 were from Sir Run Run Shaw hospital medical center. Cells were cultured in Dulbecco's modified Eagle's medium (DMEM) containing 10% fetal bovine serum (FBS), and maintained at 37°C in a humidified atmosphere with 5% CO_2_ for three days. Cells were first starved overninght with 2% serum DMEM and then changed for 10% FBS before the addition of human recombinant MSP for stimulation at a concentration of 2 nM for 5 min or DMSO as a control.

### Plasmids and transfection

Total RNA were isolated from HT29 cells using TRIzol (Life Technologies). RT-PCR was conducted by using the primer pair 1. The 700bp, 500bp and 400bp PCR fragments covering the nucleotides sequence from 1139nt to 1799nt were amplified. The 500bp fragment was then subcloned into the pGEMT-Easy vector (Promega). After confirming the deleted regions in the 500bp fragment by DNA sequencing, the cDNA fragment was digested with the restriction enzymes Eco47III and HindIII. The resulting 400bp fragment was isolated. This fragment is shorter than the regular wtRON PCR product and has an inframe deletion of 189nt. To construct the full-length RONΔ165^E2^ cDNA, the vector pGEMT-Easy RONΔ160^E2E3^ was digested with Eco47III and HindIII to obtain a 3.9kb fragment. The digested pGEMT-Easy RONΔ160^E2E3^ was then ligated with the 400bp ΔRON cDNA fragment to create the RONΔ165^E2^ cDNA(pGEMT-Easy RONΔ165^E2^). Then the mammalian expression vector pcDNA4/HisMaxC RONΔ160^E2E3^ was digested with NotI and XbaI to obtain a 5.3kb fragment. The 5.3kb fragment was then ligated with the 4.2kb RONΔ165^E2^ cDNA fragment to create the pcDNA4/HisMaxC RONΔ165^E2^. Usig transfection reagent (the FuGENE-HD from Roche) pcDNA4/HisMaxC RONΔ165^E2^, pcDNA4/HisMaxC wtRON and empty vector transfected the HEK293 cells and SW48 cells respectively. By adding 100 ug/ml Zeocin to the medium cells were selected for 2 weeks. Individual cells were cloned and analyzed the expression of variant by western blotting using the rabbit IgG to RON(C-20).

### Immunoblotting

#### Western bloting

Cells were lysed with RIPA lysis buffer (Sigma-Aldrich) containing protease and phosphatase. After quantified, cell lysates were seperated on a 7.5% SDS-PAGE, transferred to polyvinylidene difluoride (PVDF) membrane (Thermo Fisher Scientific), followed by incubation with phosphate buffered saline containing 5% milk at room temperature for 1 hours. Then the PVDF membrane was incubated overnight with primary antibodies diluted 1:1000 (while the antibody to p-tyr-100 was used at 1:2000) and horse-radish peroxidase conjugated GAPDH polyclonal antibody (Abcam) at 4°C, respectively. After washingwith TBST for 10 minutes three times, it was then incubated with goat anti-mouse or rabbit IgG (1:5000, Abcam) at room temperature for 1 hour. After washing with TBST for 10 minutes three times, the signals were detected by ECL reagents. The relative protein expression was analyzed by Image-J software 1.48V (Wayne Rasband, National Institutes of Health, USA).

#### Co-immunoprecipitation

About 700 ug cell protein lysate was diluted in IP lysis buffer and incubated with 1.5 ug normal rabbit or mouse IgG for 2h, to be followed by 2h of incubation with 15ul protein A/G plus agarose beads (Thermo Fisher Scientific, Rockford, IL, USA) to precipitate proteins that interacted non-specifically with IgG and protein A/G plus agarose. Then, the lysate was incubated with 2 ug primary immunoprecipitating antibody RON(C-20) at 4°C overnight. Protein A/G plus agarose (20ul) was added and incubated at 4°C for 6h to recover the immunocomplexes by centrifugation(1000g, 5 min at 4°C), which were then washed with IP lysis buffer (4×, 1000g, 5 min at 4°C) and proteins in the immunocomplexes were then extracted in SDS sample buffer and used for immunoblotting to identify interacting proteins. 20% of total cell lysates were subjected to western blotting with the indicated antibodies as inputs.

#### Cell surface biotinylation and immunofluorescent staining

Cell surface biotinylation was performed using a Cell Surface Protein Isolation Kit(Thermo Fisher Scientific, Rockford, IL, USA) as instructed by the supplier. Cells were first labeled with EZ Link Sulfo-NHS-SS-Biotin, athiol-cleavable amine-reactive biotinylation reagent. Cells were subsequently lysed with a mild detergent, and the labeled proteins were then isolated with NeutrAvidin Agarose. The bound proteins were released by incubating with SDS-PAGE sample buffer containing 50 mM DTT. Proteins were subjected to SDS-PAGE and Western blotting with RON(H160) antibody. The immunofluorescent staining was performed as described previously. Primary antibodies against the RON α-chain was added followed by secondary antibodies conjugated with FITC. Cells incubated with normal mouse IgG were used as controls. Sections were counter stained with DAPI. All slides were examined and photographed under Olympus BX51 upright microscope equipped with fluorescent devices.

### Migration assays

#### Scratch assay

Cells were seeded at logarithmic growth phase in 24 well plate, 5×10^^5^ each well. When the cells grew to 100% confluence, 1-2 straight lines were drawn for the cells in each well with 10ul pipette tip. Cells were cultured in each group with or without MSP (2 nM) for 24h, and then photographed for analysis. The experiments had done three times independently. The percentage of wound coverage was measured using Image-J software.

#### Transwell migration assay

The effect of wtRON or RON variant protein on MSP-negative or MSP-positive induced migration of HEK293 cells was evaluated using the Transwell migration assay. It performed by using Polyester Membrane 24 Well Transwell (8 mm, Corning, NY, USA) filters. The lower chambers of the migration filters were filled in duplicate with 600 ml of DMEM containing with or without of MSP (2 nM). Cells were plated in a volume of 100 ml 2% serum DMEM per Transwell filter at a density of 1×10^^6^. Cells were incubated in 5% CO_2_ at 37°C for 24h and were subsequently fixed by immersion of the filters in methanol at room temperature for 15 min. Filters were washed with deionized water and stained in 0.2% crystal violet in a 20% methanol/water solution for 10 min. Removed the cells from the upper surface of the membrane with a cotton swab, then counted the migrated cells underside the membrane at 200× magnification from five random fields on each membrane. The experiments done three times in the same environment and the cell numbers are mean values.

### Tumor growth *in vivo*

5-6 week old athymic female nude mice was used in our experiment. About 2×10^^6^ cells either stably transfected with RONΔ165^E2^ or wtRON were subcutaneously planted to nude mice (n=3 for each group) in the dorsal flank. Control group was subcutaneously planted with cells transfected with the blank plasmid. To accessing the bio-characteristic of the variant RONΔ165^E2^ on SW48 cells, the growth of subcutaneous-transplanted tumors was observed daily. After the formation of tumors, the tumor volume was measured every 3 days. After the tumor cells had been implanted for 30 days or the tumor had reached a volume of 1000 mm^3^, all mice were sacrificed, the tumors were peeled off. Tumor volume was measured and calculated by using the formula V(mm^3^) = 0.5×a×b^2^ (a is the maximum length todiameter and b is the maximum transverse diameter). All experiments involving mice were carried out using the recommendations of the Guide for the Care and Use of Laboratory Animals of the National Institutes of Health and were approved by the Zhejiang University Animal Care and Use Committee.

### Statistical analysis

All experiments were repeated at least three times. Data were summarized as mean ± standard error. Statistical significance was determined by student's t test or by ANOVA, using SPSS 21.0 software. A p value less than 0.05 was considered to be statistically significant.

## Abbreviations

MSP: macrophage stimulating protein
CRC: colorectal carcinoma
DMEM: Dulbecco's modified eagle medium
RT: reverse transcription
ECL: enhanced chemiluminescence
HGF: hepatocyte growth factor
